# Role of non-genomic androgen signalling in suppressing proliferation of fibroblasts and fibrosarcoma cells

**DOI:** 10.1038/cddis.2014.497

**Published:** 2014-12-04

**Authors:** G Castoria, P Giovannelli, M Di Donato, A Ciociola, R Hayashi, F Bernal, E Appella, F Auricchio, A Migliaccio

**Affiliations:** 1 Department of Biochemistry, Biophysics and General Pathology—II University of Naples, Via L. De Crecchio 7, 80138 Naples, Italy; 2 Laboratory of Cell Biology, National Cancer Institute, Bethesda, MD 20892-4256, USA; 3 Metabolism Branch, National Cancer Institute, Bethesda, MD 20892-4256, USA

## Abstract

The functions of androgen receptor (AR) in stromal cells are still debated in spite of the demonstrated importance of these cells in organ development and diseases. Here, we show that physiological androgen concentration (10 nM R1881 or DHT) fails to induce DNA synthesis, while it consistently stimulates cell migration in mesenchymal and transformed mesenchymal cells. Ten nanomolar R1881 triggers p27 Ser10 phosphorylation and its stabilization in NIH3T3 fibroblasts. Activation of Rac and its downstream effector DYRK 1B is responsible for p27 Ser10 phosphorylation and cell quiescence. Ten nanomolar androgen also inhibits transformation induced by oncogenic Ras in NIH3T3 fibroblasts. Overexpression of an AR mutant unable to interact with filamin A, use of a small peptide displacing AR/filamin A interaction, and filamin A knockdown indicate that the androgen-triggered AR/filamin A complex regulates the pathway leading to p27 Ser10 phosphorylation and cell cycle arrest. As the AR/filamin A complex is also responsible for migration stimulated by 10 nM androgen, our report shows that the androgen-triggered AR/filamin A complex controls, through Rac 1, the decision of cells to halt cell cycle and migration. This study reveals a new and unexpected role of androgen/AR signalling in coordinating stromal cell functions.

Androgens stimulate the growth of target cells, but under certain conditions slow down proliferation depending on cell type and microenvironment.^1^ Androgen binding to the androgen receptor (AR) induces differentiation of normal prostate epithelial cells and proliferation of transformed prostate epithelial cells.^2^ Additionally, AR-expressing human prostate stromal cells do not respond to androgens with proliferation.^3, 4^

Mesenchymal and transformed mesenchymal cells harbor a transcriptionally incompetent AR, and their proliferation is insensitive to physiological androgen concentration (10 nM R1881 or di-hydro-testosterone (DHT)).^5, 6^ At this concentration, these cells undergo migration as a consequence of association between AR and filamin A (FlnA).^6^ Androgen behaviors (*proliferation/migration dichotomy*) mimic those of growth factors (EGF, VEGF and PDGF), which trigger motility or proliferation depending on cell type, receptor distribution, ligand concentration and dynamic regulation of signalling networks.^7, 8, 9, 10, 11, 12^ The molecular basis of a cell's decision to ‘*go or grow'* in response to the same stimulus is not, however, completely understood.

In this study, we analyzed in NIH3T3 cells the dichotomous (proliferative/migratory) functions of AR and identified the AR/FlnA complex as the upstream player of the non-proliferative, migratory phenotype. Androgen/AR signalling initiated by the AR/FlnA complex activates Rac1, which results in cell quiescence through DYRK 1B action. Stimulation of mesenchymal cell proliferation by androgens is observed upon AR/FlnA/Rac/DYRK 1B pathway inhibition, indicating that in these cells, androgens repress the proliferative circuitry observed in classical epithelial target cells. We now report that 10 nM androgen activates the FlnA/Rac/DYRK 1B cascade to offset their growth, promoting action in mesenchymal cells. Findings in Ras-transformed fibroblasts and human fibrosarcoma HT1080 cells, harboring an activated N-Ras,^13^ strengthen the role of AR in lowering the proliferative potential of non-transformed or transformed mesenchymal cells, and suggest that new approaches are needed for the study and treatment of AR-related diseases.

## Results

### Effect of 10 nM androgens on DNA synthesis of primary, immortalized and transformed fibroblasts

**Figure 1:**
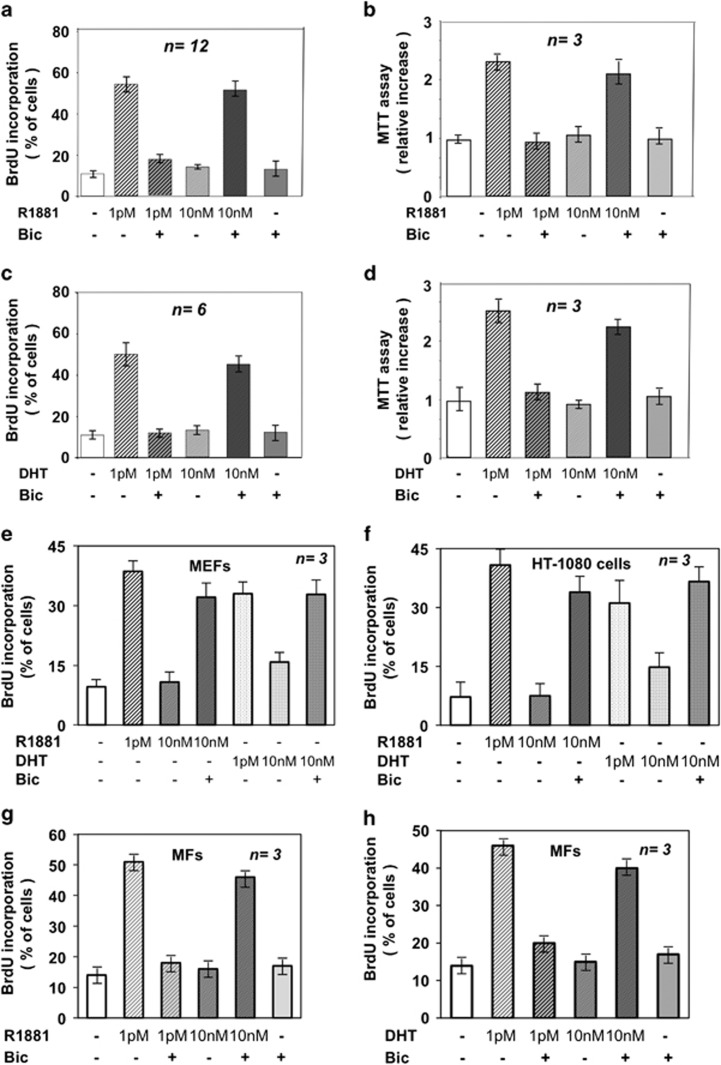
Effect of different concentrations of androgens on DNA synthesis of mesenchymal cells. Quiescent NIH3T3 cells were used and left untreated or treated with the indicated compounds. R1881 (Perkin-Elmer) or DHT (Sigma) were used at 1 pM or 10 nM; bicalutamide (Sigma-Aldrich; Bic) was added at 1000-fold excess. In (**a** and **c**), cells on coverslips were pulsed with 100 *μ*M BrdU and 18 h later BrdU incorporation was analyzed by IF. Data were expressed as % of cells. In (**b** and **d**), cell growth was measured 24 h later by MTT assay and data were expressed as relative increase. Quiescent mouse embryo fibroblasts (MEFs in **e**) or HT1080 cells (**f**) or primary mouse fibroblasts (MFs in **g** and **h**) on coverslips were left untreated or treated for 18 h with the indicated concentration of R1881 or DHT. Bicalutamide was added at 1000-fold excess. BrdU incorporation was analyzed as above and expressed as % of cells. In (**a–h**), means and S.E.M. are shown. *n* represents the number of experiments throughout the figures

NIH3T3 cells, mouse embryo fibroblasts (MEFs), primary mouse fibroblasts (MFs) and human fibrosarcoma HT1080 cells were used. In contrast to stimulation with low androgen concentration (1 picomolar; 1 pM), which has a proliferative action (Figure 1),^5^ challenging of NIH3T3 cells with 10 nM of the non-aromatizable agonist R1881 (Figure 1a) or 5*α* DHT (Figure 1c) very weakly increases BrdU incorporation in several different experiments. Stimulation of MEFs (Figure 1e), HT1080 cells (Figure 1f) or MFs (Figures 1g and h) with 10 nM R1881 or DHT does not affect DNA synthesis. Notably, the anti-androgen bicalutamide (Bic) increases BrdU incorporation, whereas it very slightly affects BrdU incorporation when added alone to the cell medium. Thus, Bic does not act on DNA synthesis by itself, but releases an inhibitory function on cell cycle progression mediated by the ligand-coupled AR. Similar results are observed using R1881 or DHT in *in vitro* cell growth assay in NIH3T3 cells (Figures 1b and d).

Ten nanomolar R1881 or DHT significantly increases motility of NIH3T3 fibroblasts (Supplementary Figure 1S A), MEFs, MFs and HT1080 cells (Supplementary Figure 2S),^5,6^ and Bic inhibits androgen-induced migration. Thus, Bic inhibits the migratory capacity of fibroblasts, while increasing their proliferative rate.

NIH3T3 fibroblasts do not express estradiol or progesterone receptor (ER or PgR; Supplementary Figures 1S B). Consistently, neither estradiol nor the synthetic progestin R5020 affects migration (Supplementary Figures 1S A), DNA synthesis (Supplementary Figures 1S C) or *in vitro* growth (Supplementary Figures 1S D).

In sum, 10 nM R1881 or DHT, which induces cell cycle progression in various AR-expressing epithelial cancer cells,^14^ does not drive DNA synthesis in primary, immortalized and transformed fibroblasts, unless they are treated with Bic.

### Ten nanomolar R1881 triggers p27 Ser10 phosphorylation and stabilization, inducing quiescence in NIH3T3 fibroblasts

**Figure 2:**
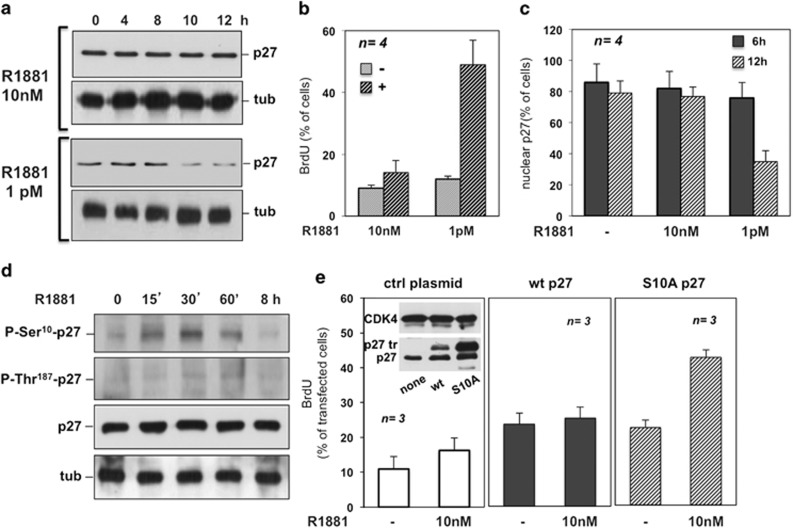
Ten nanomolar R1881-triggered p27 Ser10 phosphorylation induces quiescence. Quiescent NIH3T3 cells were used. In (**a**), quiescent cells were untreated or treated for the indicated times with 10 nM or 1 pM R1881. Lysate proteins were analyzed using anti-p27 (p27) antibody. Filters were re-probed with the anti-tubulin (tub) Ab, as a loading control. In (**b**), cells on coverslips were left untreated or treated with the indicated concentration of R1881 and pulsed with BrdU. BrdU incorporation was analyzed as above and expressed as % of cells. In (**c**), cells on coverslips were left untreated or treated for the indicated times with R1881 (10 nM or 1 pM). p27 was stained and visualized by IF. Cells showing exclusively nuclear p27 were scored and results expressed as % of cells. Data are derived from at least 600 scored cells for each experiment. In (**d**), cells were untreated or treated for the indicated times with 10 nM R1881. Lysate proteins were analyzed by western blot, using antibodies against the indicated proteins. In (**e**), cells were transfected with the empty (ctrl) or wt p27 or S10A p27 encoding plasmids. Transfected cells on coverslips were made quiescent and then untreated or treated for 18 h with 10 nM R1881. After *in vivo* pulse, BrdU incorporation was analyzed and expressed as % of transfected cells. Data are derived from at least 200 scored cells for each coverslip. Several coverslips were analyzed from three independent experiments. Inset in (**e**) shows the immunoblot of lysate proteins from cells transfected with control (none) and human p27 (wt or S10A) encoding plasmids. Lysate proteins were collected at the end of the experiment, and blotted with the antibodies against the indicated proteins. In (**b**, **c** and **e**), means and S.E.M. are shown

p27 levels are elevated in quiescent cells and decrease upon mitogenic stimulation.^15^ Ten nanomolar R1881 neither downregulates p27 (Figure 2a) nor allows DNA synthesis in NIH3T3 cells (Figure 2b). In contrast, low R1881 concentration (1 pM) decreases p27 levels (Figure 2a) and increases DNA synthesis (Figure 2b). As p27 expression might be transcriptionally regulated,^15^ we analyzed the androgen effect on luciferase reporter construct driven by the p27 promoter. Neither 10 nM nor 1 pM R1881 modifies p27 transcription in gene reporter assay, whereas serum stimulation of quiescent NIH3T3 fibroblasts increases (about fourfold) p27 transcription (Supplementary Figures 3S A). p27 is localized in the nuclei of quiescent cells and is exported in cytoplasm as the cells progress toward S-phase.^15^ Consistently, p27 is detectable in the cytoplasm of cells treated with the proliferative R1881 concentration (1 pM), whereas it remains in the nuclei of 10 nM R1881-treated cells, mimicking the results observed in quiescent cells (Figure 2c and Supplementary Figure 3S B).

Posttranslational modifications regulate p27 levels.^16^ Ser10 is one of the major phosphorylation sites of p27.^17, 18^ Challenging of quiescent NIH3T3 cells with 10 nM R1881 triggers p27 Ser10 phosphorylation, with a peak after 30 min, and slightly affects Thr187 phosphorylation in time-course experiments (Figure 2d). Quantitative analysis from three different experiments (Supplementary Figures 3S, C) shows a significant and rapid increase (about 3.5-fold within 30 min) of p27 Ser10 phosphorylation in 10 nM R1881-stimulated cells.

**Figure 3:**
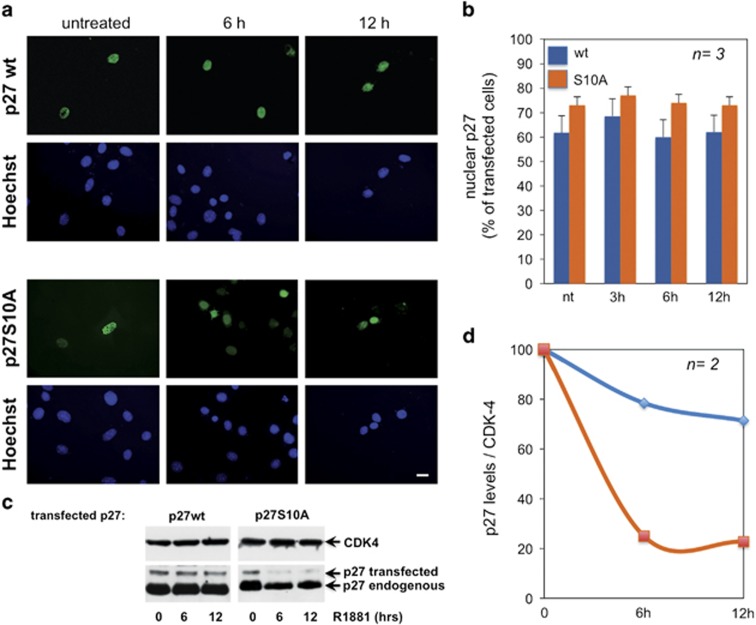
Effect of 10 nM R1881 on localization and half-life of p27 in NIH3T3 cells. NIH3T3 cells were transfected with HA-tagged wt or S10A p27. Cells were made quiescent and then left unstimulated or stimulated for the indicated times with 10 nM R1881. In (**a** and **b**), overexpressed p27 was visualized by IF as described in Methods. Panel (**a**) shows representative images from one experiment in (**b**). Bar, 10 *μ*M. Graphs in (**b**) show the quantitative score of nuclear p27, wt or S10A, overexpressed in NIH3T3 cells. Several coverslips were analyzed for each experiment, with a score of at least 100 cells for each coverslip. Means and S.E.M. are shown. In (**c** and **d**), NIH3T3 cells were transfected with HA-tagged wt p27 or S10A p27 plasmids. Cells were made quiescent and cycloheximide (50 *μ*g/ml) was added for the last 8 h. Cells were then left unstimulated or stimulated for the indicated times with 10 nM R1881. The immunoblot of lysate proteins from cells transfected with wt p27 or S10A p27 encoding plasmids is shown in (**c**). The level of transfected p27 normalized to CDK-4 from two different experiments is shown in (**d**)

Ser10 phosphorylation stabilizes p27 in G0.^19, 20^ Thus, we hypothesized that p27 Ser10 phosphorylation impairs the movement from G0 to G1-S in cells treated with 10 nM R1881. We therefore transiently transfected NIH3T3 fibroblasts with an empty plasmid or with a plasmid encoding for the human wild-type p27 (wt p27) or a mutant of p27 carrying a substitution of Ser10 with Ala (S10A p27).^17^ Both versions of p27 (wt or S10A) are overexpressed (inset in Figure 2e), with the human p27 (wt or S10A) migrating more slowly than endogenous mouse p27.^17^ Stimulation with 10 nM R1881 significantly increases BrdU incorporation in cells overexpressing the mutant S10A p27 (right panel in Figure 2e), as compared with cells overexpressing the wt p27 (central panel in Figure 2e) or the empty (left panel in Figure 2e) plasmid. Simultaneous analysis of p27 localization shows that, irrespective of transfected plasmids, p27 is localized in the nuclei of cells treated for different times (from 0 to 12 h) with 10 nM R1881 (Figures 3a and b), indicating that Ser10 phosphorylation has a negligible effect on localization of p27. Some of the nuclei expressing p27S10A are less bright (Figure 3a), likely because of the p27 mutant degradation.

Therefore, we evaluated the half-life of overexpressed p27, wt or S10A in cycloheximide-treated NIH3T3 cells. Cells were challenged with 10 nM R1881 for the indicated times. Immunoblot and quantitative analysis show that S10A p27 undergoes a rapid and robust degradation upon R1881 stimulation. In contrast, the levels of wt p27 are stable (Figures 3c and d). Cell cycle analysis by fluorescence-activated cell sorting shows that a large number of 10 nM R1881-treated NIH3T3 fibroblasts are in G0/G1 (Supplementary Figures 4S A). Thus, 10 nM R1881 triggers p27 Ser 10 phosphorylation, stabilizes p27 and maintains cell quiescence.

### Ten nanomolar R1881 activates DYRK 1B through Rac1

**Figure 4:**
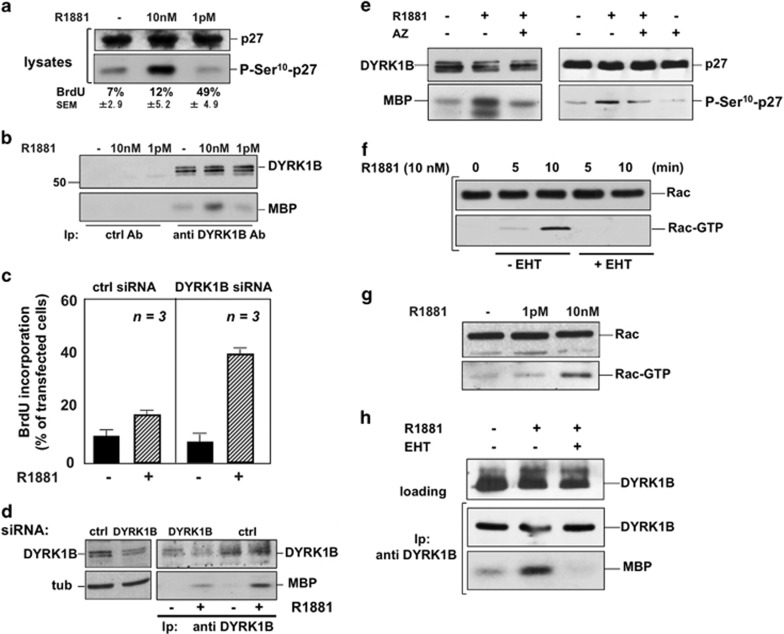
Ten nanomolar androgen activation of DYRK 1B induces quiescence through p27 Ser10 phosphorylation and is Rac-dependent. NIH3T3 cells were used. In (**a**), quiescent cells were untreated or treated for 30 min with 10 nM or 1 pM R1881. Lysate proteins were analyzed by western blot, using antibodies against the indicated proteins (lysates). The lowest section shows the corresponding BrdU incorporation analyzed by IF and expressed as % of cells. Means and S.E.M. are shown. In (**b**), quiescent cells were untreated or treated for 30 min with 10 nM or 1 pM R1881. Lysate proteins were immunoprecipitated with control (ctrl) or anti-DYRK 1B antibody. Immune complexes were analyzed for DYRK 1B activity using myelin basic protein (MBP) as a substrate. DYRK 1B levels in immune complexes were detected by immunoblot with anti-DYRK 1B antibody. Quantitative analysis of 10 nM R1881-triggered DYRK 1B activity from three different experiments was analyzed using the NIH Image J program. It showed a fivefold increase in kinase activity. In contrast, no significant increase in kinase activity was detected in cells stimulated with 1 pM R1881. In (**c**), growing cells were transfected with non-targeting siRNA (ctrl siRNA) or DYRK 1B siRNA (DYRK 1B siRNA). Cells were co-transfected with negative control siRNA Alexa-Fluor 488 to help identification of transfected cells. After transfection, cells were made quiescent, then left unstimulated or stimulated for 18 h with 10 nM R1881. After *in vivo* pulse with BrdU, BrdU incorporation was analyzed by IF and expressed as % of transfected cells. Data are derived from at least 300 scored cells for each experiment. Means and S.E.M. are shown. Lysate proteins from cells transfected with non-targeting (ctrl) or targeting (DYRK 1B) siRNA were analyzed by western blot with anti-DYRK 1B antibody (left panel in **d**), and filter was re-probed with anti-tubulin antibody, as a loading control (tub). DYRK 1B was immunoprecipitated from the same lysate proteins and its activity in immune complexes was assayed using MBP as a substrate (right panel in **d**). In (**e**), quiescent cells were left untreated or treated for 30 min with 10 nM R1881, in the absence or presence of the DYRK 1B inhibitor AZ191, which was added (1 *μ*M) 15 min before hormonal stimulation. Control cells in right panel were treated with the inhibitor alone. In left panels, lysate proteins were immunoprecipitated with anti-DYRK 1B antibody. Immune complexes were analyzed for DYRK 1B activity using MBP as a substrate. DYRK 1B levels in immune complexes were detected by immunoblot with anti-DYRK 1B antibody. In right panels, lysate proteins were analyzed by western blot, using antibodies against the indicated proteins. In (**f**), quiescent cells were left untreated or treated for the indicated times with 10 nM R1881, in the absence or presence of the Rac inhibitor EHT1864 (at 10 *μ*M), which was added 2 h before hormonal stimulation. Rac pull-down assay was performed and the amounts of total Rac and Rac-GTP were detected by western blot. In (**g**), quiescent cells were untreated or treated for 10 min with 1 pM or 10 nM R1881. Rac pull-down assay was performed as in (**f**). In (**h**), quiescent cells were untreated or treated for 30 min with 10 nM R1881 in the absence or presence of EHT1864 (10 *μ*M). Lysate proteins containing the same amount of DYRK 1B (loading) were incubated with anti-DYRK 1B antibody. The lower section shows DYRK 1B activity in immune complexes, assayed using MBP as a substrate. The upper section shows the western blot of immune complexes with anti-DYRK 1B antibody

Dual-specificity tyrosine-phosphorylation-regulated kinases (DYRKs) control proliferation and differentiation through the phosphorylation of cell cycle regulators.^21^ A member of the DYRK family kinases, DYRK 1B, induces reversible G0 arrest by stabilizing p27 through Ser10 phosphorylation.^19^ In NIH3T3 cells, there are congruent effects on DNA synthesis, as 10 nM R1881 increases p27 Ser10 phosphorylation, whereas 1 pM R1881 reduces this phosphorylation below the basal level in lysates (Figure 4a). At the same time, 10 nM R1881 increases the activity of DYRK 1B (B). In the same experiment, a weak, but not significant, increase in DNA synthesis was detected in 10 nM R1881-treated NIH3T3 cells (A). In contrast, 1 pM R1881 concentration leaves unaffected DYRK 1B activity (panel B) and robustly increases DNA synthesis (A). No increase in DYRK 1B activity is detected in cell lysates immunoprecipitated with control antibody (Figure 4b). Quantitative analysis from different experiments shows that myelin basic protein phosphorylation by immunoprecipitated DYRK 1B increased by about fivefold upon 10 nM R1881 stimulation of quiescent cells (legend to Figure 4). Thus, hormonal activation of DYRK 1B would result in P-p27-Ser10 phosphorylation and cell quiescence.

Depleting the cells of DYRK 1B increased BrdU incorporation in 10 nM R1881-treated cells (right panel in Figure 4c). BrdU incorporation in 10 nM R1881-treated control cells, expressing non-targeting siRNA, was similar to the basal level (left panel in Figure 4c). Knockdown of DYRK 1B was verified by the decrease in protein levels (left section in Figure 4d) and DYRK 1B activity (right section in Figure 4d). The DYRK 1B inhibitor AZ191^22^ almost completely blocks both androgen-triggered DYRK 1B activity (left panel in Figure 4e) and p27 Ser10 phosphorylation (right panel in Figure 4e), supporting the role of the kinase in this phosphorylation.

Small GTP-binding proteins control DYRK 1B.^23^ Ten nanomolar R1881 (Figures 4f and g), but not 1pM R1881 (Figure 4g), activates Rac1, and the Rac inhibitor EHT1864^24^ prevents R1881-triggered activation of Rac1 (Figure 4f) and DYRK 1B (Figure 4h).

In sum, Rac-dependent activation of DYRK 1B triggers p27 Ser10 phosphorylation and induces quiescence in 10 nM R118-treated cells.

### Androgen-activated Rac1 inhibits DNA synthesis and stimulates migration in fibroblasts and fibrosarcoma HT1080 cells

**Figure 5:**
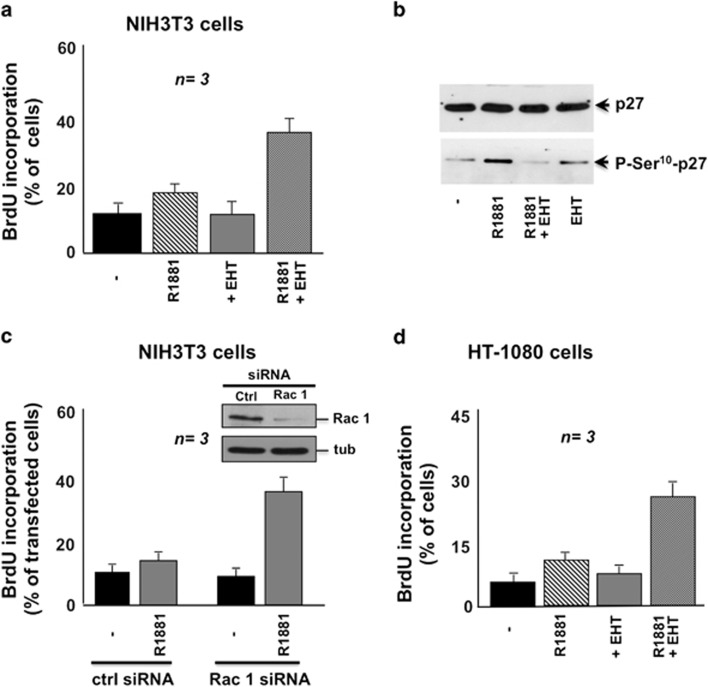
Rac inhibition triggers DNA synthesis and suppresses p27 Ser10 phosphorylation in NIH3T3 fibroblasts and human fibrosarcoma HT1080 cells treated with 10 nM R1881. In (**a**–**c**), NIH3T3 cells were used. In (**a**), quiescent cells on coverslips were left untreated or treated for 18 h with 10 nM R1881 in the absence or presence of EHT1864 (10 *μ*M). Control cells were treated with EHT1864 alone. After *in vivo* pulse, BrdU incorporation was analyzed by IF and expressed as % of cells. In (**b**), quiescent cells were left untreated or treated for 30 min with 10 nM R1881 in the absence or presence of EHT1864 (10 *μ*M). Control cells were treated with EHT1864 alone. Lysate proteins were analyzed by western blot, using antibodies against the indicated proteins. In (**c**), growing cells were transfected with non-targeting siRNA (ctrl siRNA) or Rac1 siRNA (Rac1 siRNA). Cells were co-transfected with negative control siRNA Alexa-Fluor 488 to help identification of transfected cells. After transfection, the cells were made quiescent and then left unstimulated or stimulated for 18 h with 10 nM R1881. After *in vivo* pulse, BrdU incorporation was analyzed by IF and expressed as % of transfected cells. Data are derived from at least 500 scored cells for each experiment. Inset in (**c**) shows the western blot with anti-Rac antibody of lysate proteins from cells transfected with Rac1 or non-targeting (ctrl) siRNA. The filter was re-probed with anti-tubulin antibody as a loading control (tub). In (**d**), quiescent HT1080 cells on coverslips were left untreated or treated for 18 h with 10 nM R1881 in the absence or presence of EHT1864 (10 *μ*M). Control cells were treated with EHT1864 alone. BrdU incorporation was analyzed as in (**a**). Means and S.E.M. are shown

In 10 nM R1881-treated NIH3T3 fibroblasts, EHT1864 increases DNA synthesis (Figure 5a) and inhibits p27 Ser10 phosphorylation (Figure 5b) as well as cell motility (Supplementary Figures 4S B). In cells treated with 10 nM R1881, Rac1 knockdown (inset in Figure 5c) enables BrdU incorporation, which is unaffected by non-targeting siRNA (Figure 5c). Similarly, EHT1864 increases BrdU incorporation (Figure 5d) and inhibits migration (Supplementary Figures 4S C) in 10 nM R1881-treated HT1080 cells.

In conclusion, Rac1 activation attenuates mitogenic signalling through DYRK 1B and p27 Ser10 phosphorylation, whereas it stimulates migration in 10 nM-stimulated fibroblasts and fibrosarcoma cells.

### AR/FlnA association halts DNA synthesis in androgen-treated fibroblasts

The androgen-triggered AR/FlnA complex controls Rac1 activation and motility in NIH3T3 fibroblasts.^6^ Aiming to dissect the role of this complex in regulating NIH3T3 cell proliferation, we initially looked at the effects of the overexpression of an AR mutant (Δ622-670 hAR)^25^ that does not associate with FlnA and inhibits cell motility in 10 nM R1881-treated NIH3T3 cells.^6^

**Figure 6:**
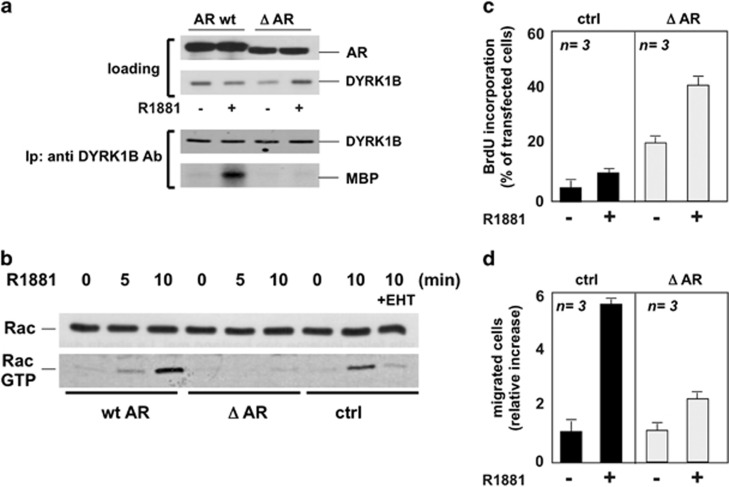
An AR mutant unable to interact with FlnA induces the proliferative phenotype and impairs motility of NIH3T3 cells challenged with 10 nM R1881. In (**a** and **b**), NIH3T3 cells were transfected with wild-type hAR (AR wt) or its mutant (622–670 hAR; ΔAR), or the empty pSG5 (ctrl) plasmid. Cells were made quiescent and then left untreated or treated for 30 min (**a**) or the indicated times (**b**) with 10 nM R1881. When indicated, EHT1864 was added at 10 *μ*M. In (**a**), AR (wt or ΔAR) and DYRK 1B were detected by western blot of lysate proteins using the appropriate antibodies (loading in upper panel). Lysate proteins were immunoprecipitated using anti-DYRK 1B antibody, and DYRK 1B activity was assayed using MBP as a substrate (lower panel). In (**b**), Rac pull-down assay was performed and the amount of Rac or Rac-GTP was detected by western blot. In (**c** and **d**), NIH3T3 cells were transfected with the empty (ctrl) or ΔAR- (ΔAR) encoding plasmid. pEGFP plasmid (at 0.2 *μ*g; Clontech) was included to help identification of transfected cells. Transfected cells were made quiescent. In (**c**), cells were left unstimulated or stimulated for 18 h with 10 nM R1881. After *in vivo* pulse, BrdU incorporation was analyzed by IF and expressed as % of transfected cells. In (**d**), cells were left unstimulated or stimulated for 6 h with 10 nM R1881. Migrated cells were scored and data expressed as relative increase. In (**c** and **d**), data are derived from at least 500 scored cells for each experiment. Means and S.E.M. are shown

The AR mutant does not mediate R1881-induced activation of DYRK 1B (Figure 6a) or Rac (Figure 6b), indicating that the AR/FlnA complex controls androgen-triggered Rac/DYRK 1B activation. Ten nanomolar R1881 challenging of cells expressing the wt hAR or the empty pSG5 control plasmid increased Rac activation (Figure 6b), which was abolished by EHT1080. Overexpression of the Δ622-670 hAR mutant increases proliferation (Figure 6c), whereas it inhibits migration in 10 nM R1881-treated NIH3T3 cells (Figure 6d).

**Figure 7:**
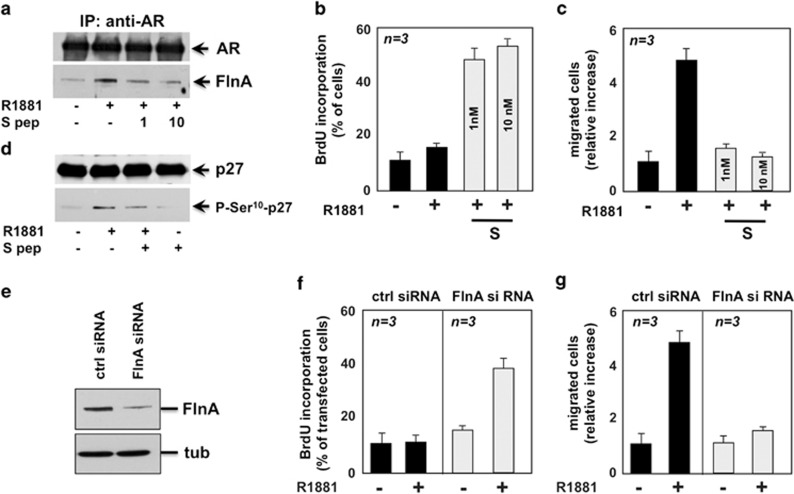
The AR/FlnA complex induces p27 Ser10 phosphorylation, stimulates migration and halts DNA synthesis in NIH3T3 cells treated with 10 nM R1881. In (**a**), quiescent NIH3T3 cells were left untreated or treated for 5 min with 10 nM R1881, in the absence or presence of the indicated concentrations of S peptide (1 or 10 nM). The peptide was added 30 min before hormonal stimulation. Lysates were incubated with anti-AR antibody and proteins in immune complexes were blotted with antibodies against the indicated proteins. In (**b**), quiescent cells on coverslips were left untreated or treated for 18 h with 10 nM R1881 in the absence or presence of the indicated concentration of S peptide. After *in vivo* pulse, BrdU incorporation was analyzed as above. In (**c**), quiescent cells were left untreated or treated with 10 nM R1881 in the absence or presence of the indicated concentrations of S peptide. Cells were allowed to migrate in trans-well chambers and migrated cells were scored by fluorescent microscopy. Data were expressed as relative increase. In (**d**), quiescent NIH3T3 cells were left untreated or treated for 30 min with 10 nM R1881, in the absence or presence of 10 nM S peptide. Control cells were treated with 10 nM S peptide alone. Lysate proteins were immune-blotted with antibodies against the indicated proteins. In (**e** and **f**), growing cells were transfected with non-targeting (ctrl siRNA) or FlnA (FlnA siRNA) siRNA. Cells were co-transfected with negative control siRNA Alexa-Fluor 488 to help identification of transfected cells. In (**e**), the western blot of lysate proteins from cells transfected with non-targeting (ctrl siRNA) or targeting (FlnA siRNA) siRNA with anti-FlnA antibody is shown. The filter was re-probed with anti-tubulin antibody as the loading control (tub). In (**f**), cells transfected with ctrl siRNA or FlnA siRNA were made quiescent and then left unstimulated or stimulated for 18 h with 10 nM R1881. After *in vivo* pulse, BrdU incorporation was analyzed by IF and expressed as % of transfected cells. In (**g**), cells transfected with ctrl siRNA or FlnA siRNA were made quiescent and then left unstimulated or stimulated with 10 nM R1881. Migrated cells were scored and data expressed as relative increase. In (**b**, **c**, **f** and **g**), data are derived from at least 500 scored cells for each experiment. Means and S.E.M. are shown

As the hAR mutant lacks a large amino acid sequence, we synthesized a small peptide derived from the AR 622-670 amino acid sequence responsible for interaction of the receptor with FlnA.^25^ A hydrocarbon-stapled version of this peptide was also synthesized in which residues Ala628 and Lys632 were each replaced with an olefinic amino acid that allows them to be cross-linked. This cross-link locks the peptide into an alpha-helical conformation, as this sequence is predicted to form an alpha helix. The stapled version should have increased activity. In fact, stapled peptides have increased cell permeability, higher affinity for the target, and decreased degradation as compared to their unstapled counterpart.^26^ The amino acid sequence of the stapled peptide (S) is shown in Supplementary Figure 5S. The S peptide displaces the 10 nM R1881-induced AR/FlnA complex assembly in NIH3T3 cells (Figure 7a) and inhibits p27 Ser10 phosphorylation (Figure 7b). Again, it increases DNA synthesis (Figure 7c) and inhibits cell motility (Figure 7d) triggered by 10 nM R1881 in NIH3T3 cells. Furthermore, the S peptide does not affect serum-induced DNA synthesis or motility in NIH3T3 cells, and does not interfere with serum-induced DNA synthesis and motility in AR-negative human prostate cancer DU-145 cells (Supplementary Figures 5S, A–D). Thus, the S peptide specifically acts in androgen-stimulated AR-expressing cells. Finally, the S peptide does not disrupt the well-known association between FlnA and integrin *β*1 ^27^ in NIH3T3 cells (Supplementary Figure 5S E) and leaves unaffected AR-mediated transcriptional activity in NIH3T3, LNCaP and HT1080 cells (Supplementary Figure 5S F).

Depleting NIH3T3 cells of FlnA (Figure 7e) increases DNA synthesis (Figure 7f) and inhibits migration (Figure 7g) triggered by 10 nM R1881.

In conclusion, data in Figures 6 and 7 indicate that the AR/FlnA complex simultaneously halts cell cycle and promotes migration in cells challenged with physiological androgen concentration through its action on Rac. Interfering in this complex restores the proliferative phenotype, whereas impairing the migratory phenotype of fibroblasts.

### Androgens counteract Ras-induced transformation

**Figure 8:**
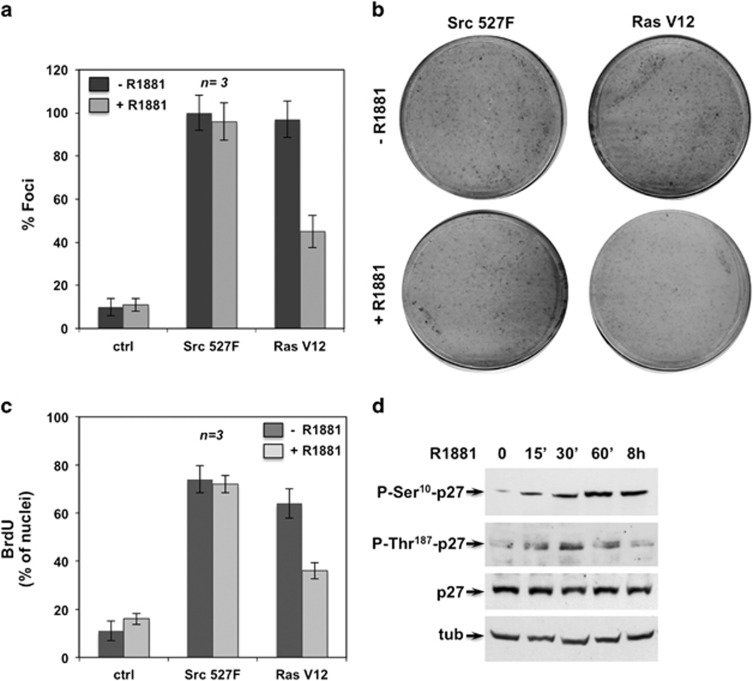
Androgen inhibits Ras- but not Src-induced transformation. In (**a** and **b**), NIH3T3 cells were transfected with the indicated expression plasmids or the empty plasmid (ctrl) and cultured in the absence or presence of 10 nM R1881 as reported in Methods. After 12 days, the cells were stained with 0.5% crystal violet and the number of foci was counted. In (**a**), mean values from three independent experiments, each conducted in duplicate, are shown in the graph, representing the percentages of transformed foci relative to Src527F or RasV12 plasmids. The standard error for each value is shown. *n* represents the number of experiments. In (**b**), representative plates from a single experiment conducted in duplicate are shown. Growing Src- or Ras-transformed NIH3T3 cells were used. In (**c**), cells on coverslips were maintained for 3 days in 0.5% dextran-coated charcoal-stripped serum and then left unstimulated or stimulated for 18 h with 10 nM R1881. After *in vivo* pulse, BrdU incorporation was analyzed as above. Data are derived from at least 800 scored cells for each experiment. Means and S.E.M. are shown. *n* represents the number of experiments. In (**d**), Ras-transformed cells were maintained for 3 days in 0.5% dextran-coated charcoal-stripped serum and then left unstimulated or stimulated for the indicated times with 10 nM R1881. Lysate proteins were analyzed using the anti-P-Ser10 or P-Thr197 p27. Filters were re-probed with p27 or anti-tubulin (tub) Ab, as the loading control

Ras- and Src-transformed NIH3T3 fibroblasts harbor AR (Supplementary Figure 2S B).^5^ We therefore analyzed the ability of androgens to reduce fibroblast transformation induced by the oncogenic form of Src (Src527F)^28^ or Ras (V12Ras).^29^ Albeit to a different extent, the activated forms of Src and Ras transform NIH3T3 fibroblasts in a focus assay (Figures 8a and b). Ten nanomolar R1881 drastically reduces transformation by Ras, leaving Src-induced transformation unaffected (Figures 8a and b). Again, 10 nM R1881 inhibits BrdU incorporation in Ras- but not in Src-transformed fibroblasts (Figure 8c), and triggers rapid (15 min) and persistent (8 h) p27 Ser10 phosphorylation in Ras-transformed cells. In contrast, p27 Thr187 phosphorylation peaks at 30 min and then decreases (Figure 8d). The sustained p27 Ser10 phosphorylation we observe in different experiments (Supplementary Figures 6S A) is likely due to DYRK 1B hyperactivation in Ras-transformed cells.^30^ Thus, the rapid and persistent p27 Ser10 phosphorylation induced by androgens in Ras-transformed fibroblasts overcomes the effect of p27 Thr187 phosphorylation with consequent p27 degradation,^31^ and might be responsible for the inhibitory action of androgen. Oncogenic Src likely escapes the androgen effect through alternative nuclear effectors (e.g., Myc)^28^ to those used by Ras in transforming cells.

## Discussion

We here investigated the role of AR in proliferation of mesenchymal and transformed mesenchymal cells. Ten nanomolar R1881 or DHT fails to induce DNA synthesis in primary or immortalized untransformed fibroblasts and human fibrosarcoma HT1080 cells. The anti-androgen Bic (Casodex) allows DNA synthesis and proliferation, indicating the involvement of AR in these proliferative responses to the antagonist. It should be noted that Bic, frequently used in human PCa androgen deprivation therapy, often promotes PCa progression.^1^ Our findings indicate that Bic induces undesired signals in mesenchymal cells.

Androgens stimulate fibroblast DNA synthesis at very low (picomolar) concentration, and cell motility at higher (nanomolar) concentration.^5, 6^ Thus, mesenchymal cells can switch from a proliferative to a migratory phenotype when androgen concentration increases. The migratory effect exerted by androgens could play a role in different physiological and pathological conditions, including male sexual differentiation, when presumptive peritubular myoid cells migrate into the testis from mesonephros. At this stage, peritubular myoid cells strongly reduce their proliferation rate and begin to consistently express AR during their inward migration.^32^ In adults, such a switch might locally occur during wound healing or cancer progression.

We analyzed the mechanism underlying the molecular switch from proliferative to migratory action of androgens. The mode of action of androgens does not depend on the dichotomous androgen effect on AR-mediated transcription or AR nuclear translocation, as shown in Supplementary data (Supplementary Figure 6S).^5^ AR is, indeed, permanently poised in the extra-nuclear compartment of NIH3T3 and HT1080 cells, where it recruits different effectors or scaffolds leading to different biological outcomes. At low androgen levels, AR recruits Src and the regulatory subunit of PI3-K, and activates Erk and Akt (Supplementary Figure 6S),^5^ thereby fostering proliferation. In contrast, at high androgen levels, AR recruits FlnA and cell migration follows.^6^ Upon 10 nM androgen-triggered AR/FlnA complex assembly, FlnA acts as a scaffold for the spatial organization of the Rac-mediated signalling pathway, enabling the recruitment by Rac of specific effectors (e.g., DYRK 1B) involved in cell quiescence.

In addition to stimulating proliferation, Rho protein family members might inhibit cell cycle.^33^ Rho E behaves as an onco-suppressor gene in PCa^34^ and halts cell cycle in serum-stimulated NIH3T3 fibroblasts.^35^ We here report that AR-mediated cell cycle inhibition depends on the activation of Rac 1 and its dependent pathway in fibroblasts and HT1080 cells. DYRK 1B is controlled by Rac-activated MKK3 and induces NIH3T3 cell quiescence.^19^ We have consistently observed that 10 nM R1881 triggers Rac activity, which controls DYRK 1B in NIH3T3 cells. Experiments with inhibitors of Rac1 and DYRK 1B strengthen the role of these effectors in androgen-driven p27 Ser10 phosphorylation. Neither Rac nor DYRK 1B activation is detected in NIH3T3 cells treated with low (1 pM) androgen concentration, which triggers the proliferative phenotype. Silencing and chemical inhibition of DYRK 1B allows DNA synthesis, further supporting the role of this enzyme in the quiescent state of 10 nM R1881-treated NIH3T3 fibroblasts. The observed effect by EHT1864 or Rac silencing links androgen-triggered Rac activation with DYRK 1B activity and cell quiescence. In sum, the 10 nM androgen-triggered FlnA/Rac/DYRK 1B pathway halts cell cycle through p27 Ser10 phosphorylation and might also lead to the inhibition of transformation induced by oncogenic Ras, but not oncogenic Src in these cells. Thus, AR could act as a growth suppressor by specifically inhibiting Ras-driven pathways, without interfering in the proliferation elicited by different signalling mechanisms. DYRK 1B is an active kinase in pancreatic, ovarian and colon cancer cells, and controls hedgehog signalling in the stromal compartment of mouse model of pancreatic cancer.^36^ Oncogenic Ras activates DYRK 1B through the Rac1/MKK3 signalling pathway, indicating that DYRK 1B acts as a regulator in Ras-driven transformation and tumor progression.^30, 36, 37^ Notably, DYRK 1B inhibition enables the growth of stromal cells in a genetic model of pancreatic cancer, likely through hedgehog signalling.^37^ By increasing fibroblast growth and stromal collagen content, DYRK 1B inhibition might restrain tumor growth *in vivo*.

Ras oncogenic mutations have been described in a large number of cancers, including pancreatic (90%), colon (50%), lung (30%) and thyroid tumors (50%) as well as myeloid leukemia (30%).^38^ Pancreatic, colon and lung cancers express AR.^39, 40^ Proliferation of various AR-expressing pancreatic cancer cells is insensitive to DHT,^41^ and androgens inhibit colon tumor survival through a putative membrane AR.^42^ The growth of small-cell lung carcinoma H1184 cell line is sensitive to low, but not high, DHT concentrations, and the growth of non-small-cell lung carcinoma H1993 cell line is inhibited by DHT.^39^ We observe that the proliferation of human fibrosarcoma HT1080 cells, exhibiting an activated N-Ras,^13^ is insensitive to 10 nM androgen. Thus, androgens might elicit anti-proliferative signals in human cancers bearing Ras oncogenic mutations. Altogether, these findings indicate that further exploration of the androgen/AR axis should be pursued in stromal and epithelial compartment of these cancers. Lastly, the stapled peptide used in this study, as well as other small molecules capable of disrupting the AR/FlnA interaction, might represent a promising approach to specifically modulate AR functions in stromal tissue and rescue hormone proliferative responsiveness in human prostate cancers.

## Materials and Methods

### Chemical reagents

TentaGel R RAM resin was obtained from Peptide International Inc. (Louisville, KY, USA). N-alpha-Fmoc-protected amino acids were purchased from Novabiochem (San Diego, CA, USA). The N-alpha-Fmoc-protected unusual olefinic amino acid (S)-2-(((9H-fluoren-9-yl) methoxy)carbonyl-amino)-2-methyl-hept-6-enoic acid (Fmoc-S5-OH) was used for synthesis of the stapled peptide. Solvents and coupling reagents, 2-(1H-benzotriazole-1-yl)-1,1,3,3-tetramethyluronium hexafluorophosphate and 1-hydroxybenzotriazole were obtained from Sigma-Aldrich (Milwaukee, WI, USA) and American Bioanalytical (Natick, MA, USA). The Rac inhibitor EHT1846 was from Sigma. The DYRK 1B inhibitor AZ191 was from Selleckchem (Houston, TX, USA).

### Constructs

The wt hAR was in pSG5 ^43^ and the mutant Δ622-670 hAR was in psV1.^25^ The kinase-active Src (Src527F)^28^ and the active form of Ras (Ras V12)^29^ were used. The HA-tagged wt p27 and the mutant Ser10Ala p27 in pcDNA3^17^ were a gift from M. Pagano (Department of Pathology, NYU Cancer Institute, University School of Medicine and Howard Hughes Medical Institute, NY, USA). The human full-length p27 promoter (pGL2-p27-Luc) was a gift from P. Coffer (Department of Immunology, University Medical Center Utrecht, Utrecht, The Netherlands). The 3416 construct, containing four copies of the wt *slp*-HRE2 (5′-TGGTCAgccAGTTCT-3′) and the 3424 construct (5′-TGGACAgccAGTTCT-3′), were cloned in the Nh*e*I site in pTK-TATA-Luc.^44^

### Cell culture, transfection, transactivation assay and siRNA

Src- and Ras-transformed NIH3T3 fibroblasts were a gift from M.V. Barone (Department of Translational Medical Science and European Laboratory for the Investigation of Food Induced Disease, University of Naples Federico II, Naples, Italy). They were cultured in Dulbecco's modified Eagle's medium (DMEM) supplemented with 10% fetal calf serum (FCS), 100 U/ml penicillin, 100 *μ*g/ml streptomycin (Pen/Strepto) and glutamine (2 mM), and maintained at 37 °C in humidified 5% CO_2_ atmosphere. Twenty-four hours before androgen stimulation, cell medium was replaced with phenol red-free DMEM containing Pen/Strepto and supplemented with 0.1% charcoal-stripped serum. Karyotypically heterogeneous human fibrosarcoma HT1080 cells^45^ were a gift from P. Friedl (Department of Cell Biology, Radboud University Nijmegen Medical Centre, Nijmegen, The Netherlands). HT1080 cells, early-passage NIH3T3 cells, MEFs and MFs were cultured in DMEM supplemented with 10% FCS, Pen/Strepto, glutamine (2 mM), and maintained at 37 °C in humidified 5% CO_2_ atmosphere. Unless otherwise stated, NIH3T3, MEF, MF and HT1080 cells were made quiescent using phenol red-free DMEM supplemented with Pen/Strepto, glutamine (2 mM) and 0.1% charcoal-stripped FCS for 24 h. Prostate cancer-derived fast-growing LNCaP and DU-145 cells were from ATCC (Manassas, VA, USA). They were cultured in RPMI1640 supplemented with 5% (LNCaP) or 10% (DU-145) FCS and Pen/Strepto, and maintained at 37 °C in humidified 5% CO_2_ atmosphere. After reaching sub-confluence, growing LNCaP cells were kept in phenol red-free RPMI1640 medium containing 5% charcoal-stripped calf serum and Pen/Strepto for 3 days and then used. Growing DU-145 cells were kept in phenol red-free RPMI1640 containing Pen/Strepto and glutamine (2 mM) in the absence of serum for 24 h and then used. Purified wt or ΔAR mutant encoding plasmids were transfected at 1 *μ*g in sub-confluent NIH3T3 cells, using the Superfect reagent (QIAGEN GmbH, Hilden, Germany). Twenty-four hours later, transfected cells were made quiescent and used. For ARE-luc reporter assay, NIH3T3 cells (at 70% confluence) in phenol red-free DMEM containing 10% charcoal-stripped serum were used. Cells were transfected by Superfect with 1 *μ*g of 3416-pTK-TATA-Luc or 3424-pTK-TATA-Luc constructs, alone or with 0.5 *μ*g pSG5-hAR-expressing plasmid. Ten hours later, transfected cells were made quiescent for 24 h and then left unstimulated or stimulated with 1 pM or 10 nM R1881 (Perkin Elmer, Waltham, MA, USA) for 18 h. The purified human p27 promoter was transfected (at 1 *μ*g) using Superfect in sub-confluent NIH3T3 cells in phenol red-free DMEM containing 10% serum. Twelve hours later, transfected cells were made quiescent and then left unstimulated or stimulated with 1 pM or 10 nM R1881 (Perkin Elmer) or 20% serum for 10 h. Luciferase activity from cell lysates was measured using a luciferase assay system (Promega Corporation, Madison, WI, USA) and values were corrected using CH110-expressed-*β*-galactosidase activity (GE Healthcare, Little Chalfont, UK). The purified human HA-tagged p27 (wt or S10A) was transfected (at 0.5 *μ*g) using Superfect in sub-confluent NIH3T3 cells cultured in DMEM containing 10% serum. Ten hours later, transfected cells were made quiescent for 36 h and then left unstimulated or stimulated. When indicated, cycloheximide (Sigma) was added (at 50 *μ*g/ml) 8 h before hormonal stimulation. FlnA siRNA was performed using a three target-specific 20–25 nt siRNA pool (Santa Cruz Biotechnology, Inc., Dallas, TX, USA). Rac1 siRNA was performed using a four target-specific 20–25 nt siRNA pool (Santa Cruz). Non-targeting siRNAs, containing a scrambled sequence, were from Santa Cruz. DYRK 1B siRNA was performed using double-stranded Stealth DYRK 1B siRNA (Explera, Jesi, Italy). Non-targeting siRNA was from Explera. siRNAs were transfected using Lipofectamine 2000 (Gibco, Monza, Italy) in growing NIH3T3 cells in Optimem/DMEM (50% v/v) containing 10% serum. Transfection medium was discarded 10 h later and cells were washed twice using phenol red-free DMEM. Transfected cells were then made quiescent for 36 h and used.

### Migration, DNA synthesis, MTT analysis, fluorescence-activated cell sorting analysis and transformation assay

Trans-well assay was performed using collagen (type I from rat-tail at 100 mg/ml; BD Biosciences, San Jose, CA, USA)-coated Trans-well chamber system with 8 *μ*m pore polycarbonate membrane (Nunc, Thermo Fisher Scientific Inc., Waltham, MA, USA). NIH3T3 cells were plated in the upper chamber at 2 × 10^4^ per well in 200 *μ*l of phenol red-free DMEM containing 0.5% bovine serum albumin. In FlnA siRNA experiments, NIH3T3 cells were co-transfected with siRNA Alexa Fluor 488 to help identify the transfected cells. Cells were allowed to migrate for 6 h in a humidified chamber at 37 °C with 5% CO_2_ in the absence or presence of the indicated compounds. Cells on the upper side were then detached. NIH3T3 fibroblasts on the underside were fixed in 4% paraformaldehyde (Sigma) for 15 min and stained with Hoechst 33258 (Sigma) for 10 min. Alexa Fluor 488-transfected NIH3T3 fibroblasts were fixed in 4% paraformaldehyde for 15 min. Cells were finally counted with a DMBL (Leica, Wetzlar, Germany) fluorescent microscope using HCPL Fluotar × 20 objective in 10 random microscopic fields. DNA synthesis was evaluated by analysing BrdU incorporation. After *in vivo* pulse with 100 *μ*M (final concentration) BrdU (Sigma), BrdU incorporation was analyzed by immunofluorescence (IF), as reported.^5^ MTT assay was performed as described.^46^ For fluorescence-activated cell sorting analysis, quiescent NIH3T3 cells (2.0 × 10^5^ for each experimental point) were left untreated or treated for the indicated times with 10 nM R1881. Cells were collected, resuspended in 500 *μ*l of a buffer containing 0.1% NP-40, 0.1% sodium citrate, 50 *μ*g/ml propidium iodide, 1 mg/ml RNAse A and 0.1 mM EDTA. Cells were then incubated in the dark for 30 min and samples were measured by a FACS Calibur flow cytometer using Cell Quest software (Becton Dickinson, BD Biosciences). Results from three different experiments were analyzed using Cell Quest software (Becton Dickinson) and ModFit LT version 3 Software (Verity, Topsham, ME, USA). For transformation assay, early-passage NIH3T3 cells were seeded at 1.5 × 10^5^ cells per well in 6-well dishes the day before transfection with purified RasV12 or Src527F encoding plasmids (both at 0.5 *μ*g). Transfection was performed using Superfect in cells cultured in DMEM containing 10% charcoal-stripped FCS, as reported.^47^ Two days later, the cells were transferred to 10 cm plates. After reaching confluence, they were kept in DMEM containing 5% charcoal-stripped FCS. When indicated, R1881 (10 nM) was added every day. Control plates were treated with vehicle alone (0.001% ethanol). After 12 days, the plates were stained with 0.5% crystal violet.

### Immunofluorescence

Cells on coverslips were fixed and permeabilized.^5^ Endogenous AR in NIH3T3 cells was visualized using diluted (1 : 100 in PBS) rabbit polyclonal anti-AR antibody (Ab-2, Neo-Markers, Thermo Fisher Scientific Inc.). Rabbit antibody was detected using diluted (1 : 200 in PBS containing 0.2% bovine serum albumin) anti-rabbit fluorescein-conjugated antibodies (Jackson Laboratories, Las Vegas, NV, USA). DNA synthesis was analyzed using diluted (1 : 50 in PBS) mouse monoclonal anti-BrdU antibody (clone BU-1, GE Healthcare). Mouse antibody was detected using diluted (1 : 200 in PBS) Texas red-conjugated goat anti-mouse antibody (Jackson Laboratories). Endogenous p27 was stained using diluted (1 : 100 in PBS containing 0.1% bovine serum albumin) rabbit anti-p27 polyclonal antibody (C-19; Santa Cruz). Diluted (1 : 200 in PBS) anti-rabbit fluorescein isothiocyanate-conjugated antibody (Jackson Laboratories) was used as a secondary antibody. For localization of ectopically expressed p27, staining was performed using anti-HA (F-5; Santa Cruz) as primary antibody and FITC-conjugated anti-mouse IgG (Jackson Laboratories) as the secondary reagent. Coverslips were finally stained with Hoechst 33258, inverted and mounted in Mowiol (Calbiochem, Millipore/Merck KGaA, Darmstadt, Germany). Fields were analyzed with a DMBL Leica (Leica) fluorescent microscope using HCX PL Apo × 63 oil objective. Images were captured using DC480 camera (Leica) and acquired using FW4000 (Leica) software. Confocal microscopy analysis was performed using a Zeiss LSM 510 laser scanning confocal microscope (Carl Zeiss Microscopy GmbH, Göttingen, Germany), as reported.^48^ The image collection periods and exposures are identical for the different experimental conditions.

### Peptide synthesis

Peptides were synthesized by 9-fluorenylmethoxycarbonyl (Fmoc)/tert-butyl solid-phase method with an Applied Biosystem (Thermo Fisher Scientific Inc.) peptide synthesizer A431. Peptides were assembled on TentaGel R RAm resin with an initial load of 0.19 mmol/g. Coupling reactions were conducted by means of the 2-(1H-benzotriazole-1-yl)-1,1,3,3-tetramethyluronium hexafluorophosphate-1-hydroxybenzotriazole method. N-terminal primary amines were acetylated utilizing acetic anhydride and diisopropylethylamine (DIEA). For the stapled peptide, Fmoc-S5-OH was coupled manually in threefold molar excess in the presence of 2-(1H-benzotriazole-1-yl)-1,1,3,3-tetramethyluronium hexafluorophosphate, 1-hydroxybenzotriazole and DIEA. The ring-closing metathesis reaction was performed on the N-terminal acetylated peptide while still on the solid support according to literature.^49, 50, 51^ Cleavage of peptides from the resin was achieved with a trifluoroacetic acid/water/triisopropylsilane mixture (92.5/5/2.5, v/v) for 3 h. After the resin had been removed by filtration, the filtrate was concentrated by flushing with nitrogen gas and crude peptides were precipitated with diethyl ether. Crude peptide was purified using reversed-phase high-performance liquid chromatography on a preparative C4 column (BioAdvantage Pro 300, Thomson Liquid Chromatography, Thomson Instrument Company, Oceanside, CA, USA) with a water/acetonitrile solvent system containing trifluoroacetic acid. Purified peptide was characterized by matrix-associated laser desorption ionization time-of-flight mass spectrometry (MALDI micro MX, Waters,Milford, MA, USA) and reversed-phase high-performance liquid chromatography on an analytical C18 column (Eclipse XDB-C18, Agilent Technology, Santa Clara, CA, USA). The purity of the peptides was found to be >95%.

### Antibody, immunoprecipitation, immunoblotting, kinase and Rac assays

Lysates (at 2 mg/ml protein concentration) were prepared as reported.^14^ Rac activity from quiescent NIH3T3 was analyzed as described,^5^ using the Rac Activation Assay Kit (Upstate Biotechnology Inc., Lake Placid, NY, USA). AR was revealed using rabbit polyclonal anti-AR antibodies (N-20 or C19; Santa Cruz). The rabbit polyclonal anti-ERalpha antibody (543; Santa Cruz) was used to detect ERalpha. PgR was detected using the mouse monoclonal anti-PgR antibody (6A1; Cell Signaling Technology, Danvers, MA, USA). Total Akt and P-Ser 473 Akt were detected using appropriate antibodies (Cell Signaling Technology). Erk and P-Tyr 204 Erk were detected using appropriate antibodies (Santa Cruz). p27 was detected using rabbit anti-p27 polyclonal antibody (C-19; Santa Cruz; diluted 1 : 100 in PBS containing 0.1% bovine serum albumin). For CDK-4 detection, the rabbit polyclonal antibody (c-22 from Santa Cruz) was used. Rabbit polyclonal anti-phosho-p27 antibodies (P-Ser10 or P-Thr187; Zymed Laboratories, Thermo Fisher Scientific Inc.) were used to reveal P-p27. DYRK 1B was immunoprecipitated and revealed by western blot using the rabbit polyclonal anti-DYRK 1B antibody (Cell Signaling Technology). DYRK 1B activity in immune complexes was assayed as reported,^52^ using myelin basic protein as a substrate. The ECL system (GE Healthcare) was used to detect immunoreactive proteins.

## Abbreviations

AR: androgen receptor
BrdU: bromodeoxyuridine
DHT: dihydrotestosterone
ER: estradiol receptor
ECM: extracellular matrix
IF: immunofluorescence
DYRK 1B: dual-specificity tyrosine-phosphorylation-regulated kinase 1B
MAPK: mitogen-activated protein kinase
MEK-1: mitogen-activated kinase
MKK3: MAP kinase 3
PgR: progesterone receptor
