# Supplementary figures and images for: Role of non-genomic androgen signalling in suppressing proliferation of fibroblasts and fibrosarcoma cells
Source: Cell Death Dis. 2014 Dec 4;5(12):e1548–. doi: 10.1038/cddis.2014.497 (PMC4649827; doi:10.1038/cddis.2014.497)

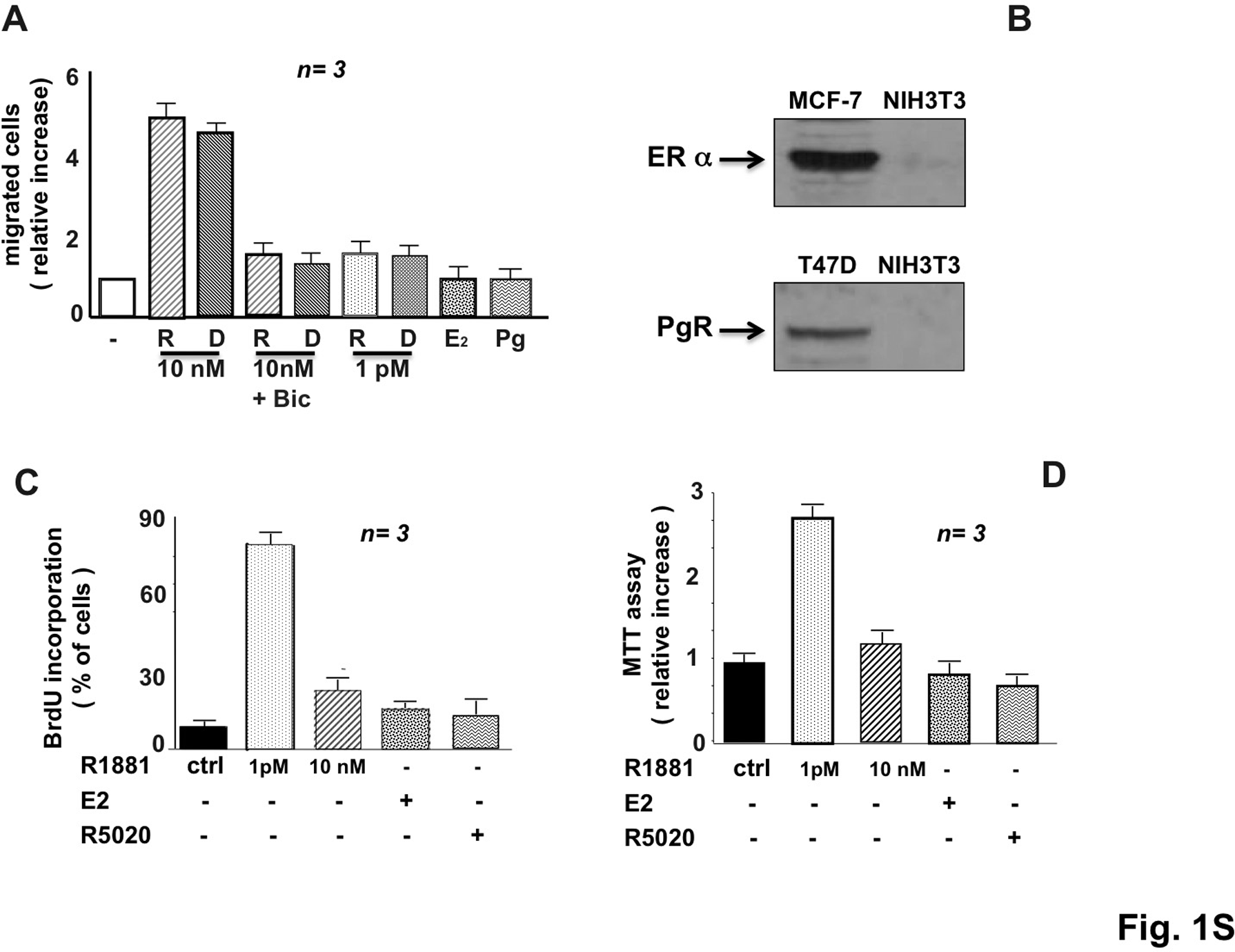

Supplement: Supplementary Figure S1 [file cddis2014497x1.tif]

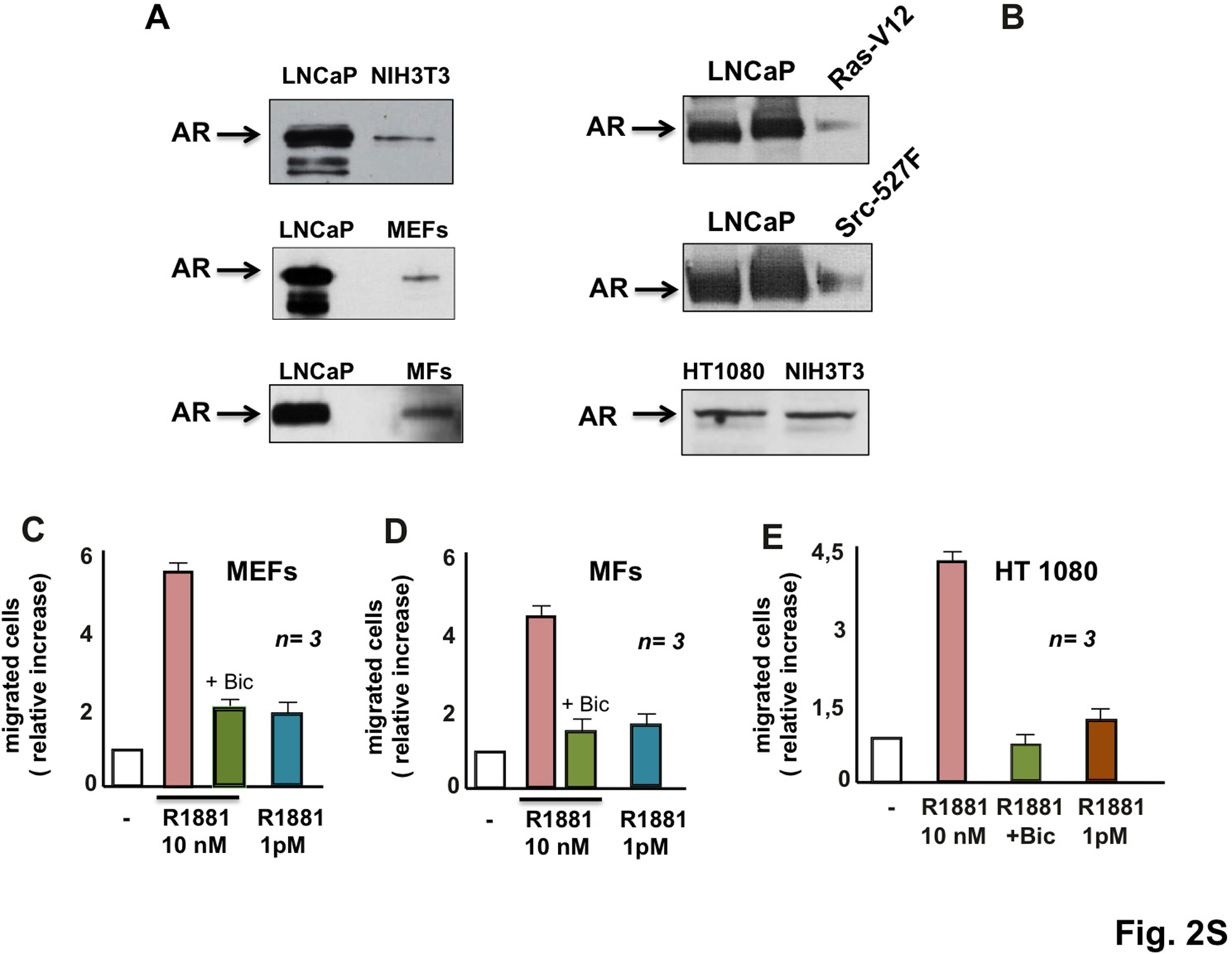

Supplement: Supplementary Figure S2 [file cddis2014497x2.tif]

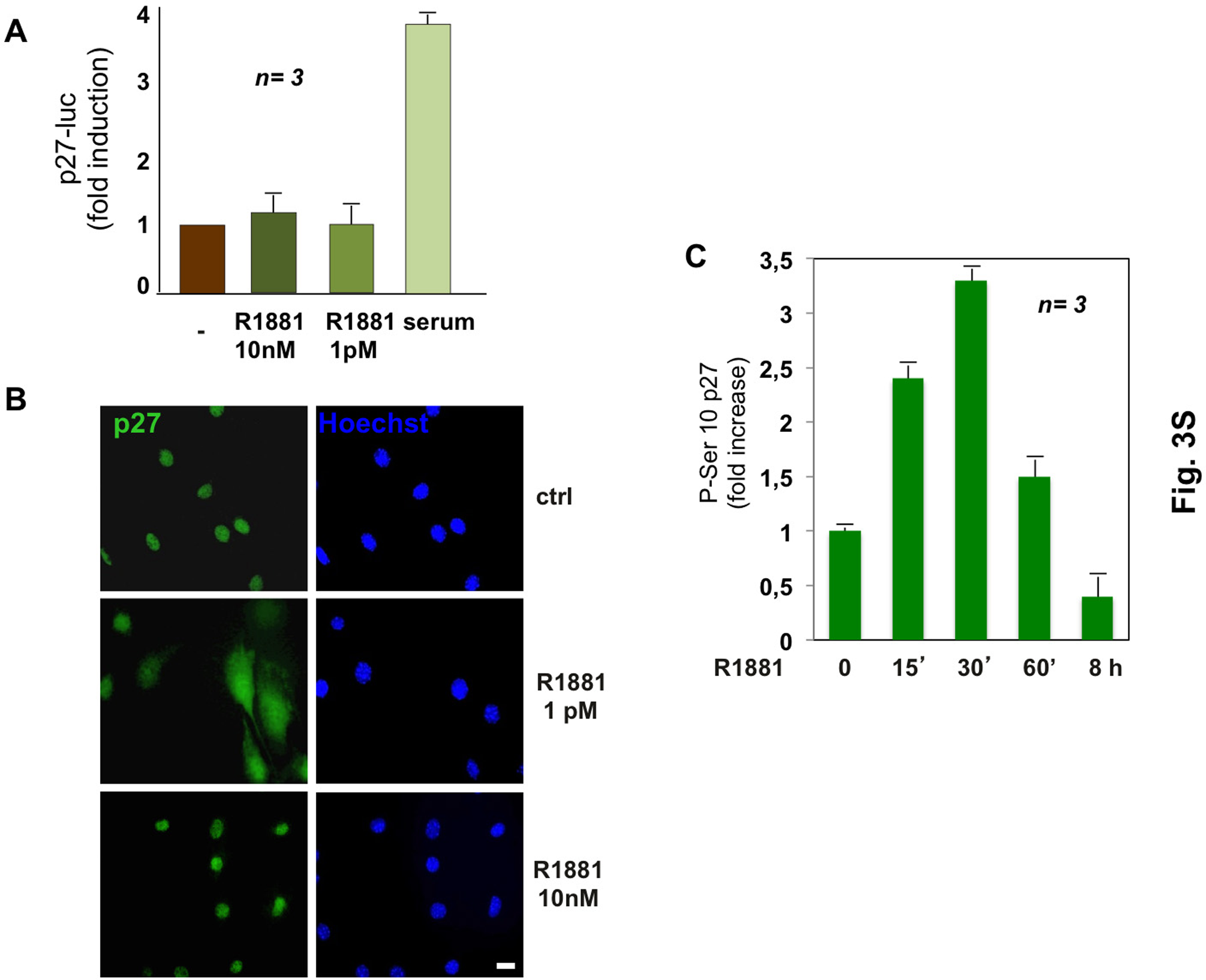

Supplement: Supplementary Figure S3 [file cddis2014497x3.tif]

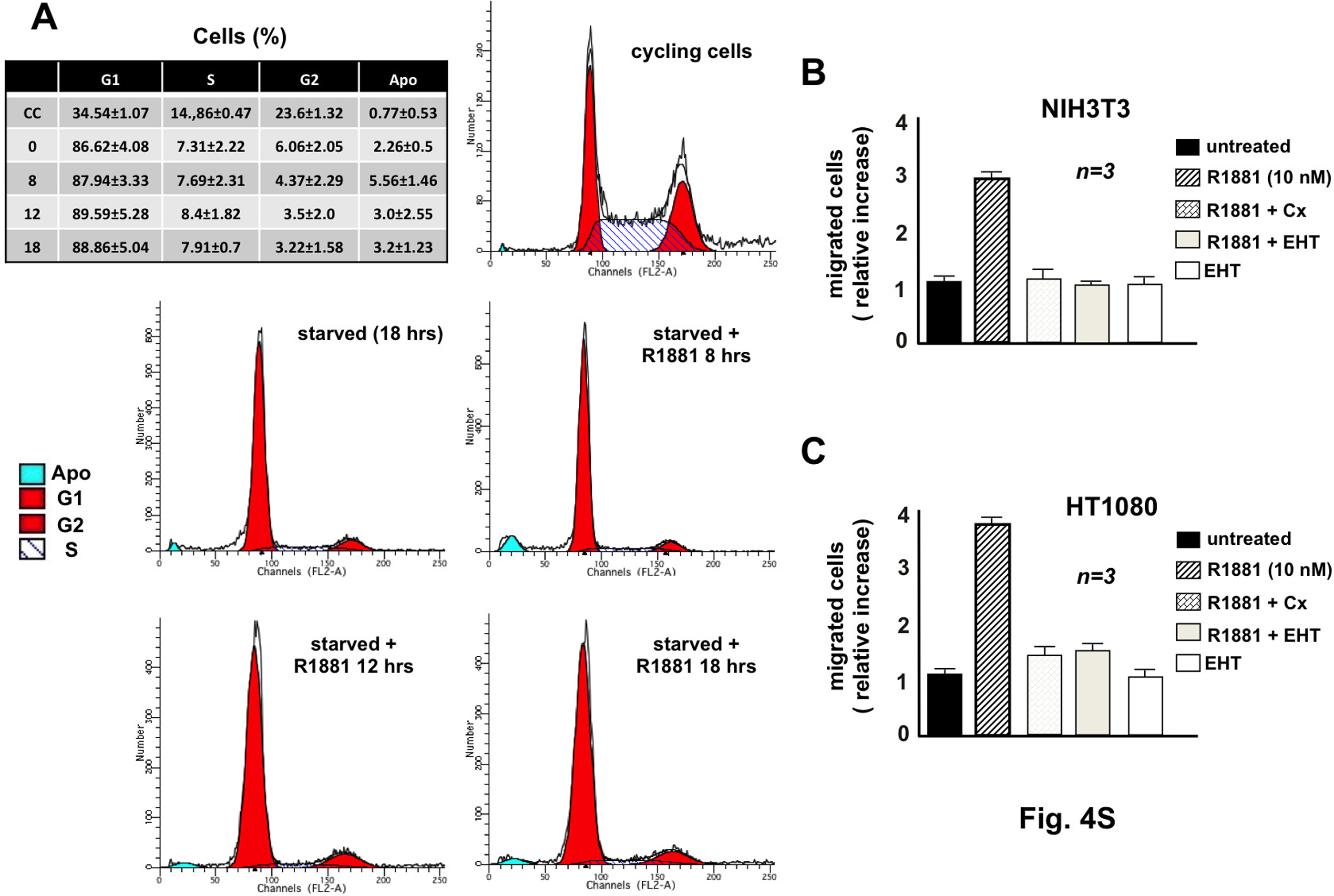

Supplement: Supplementary Figure S4 [file cddis2014497x4.tif]

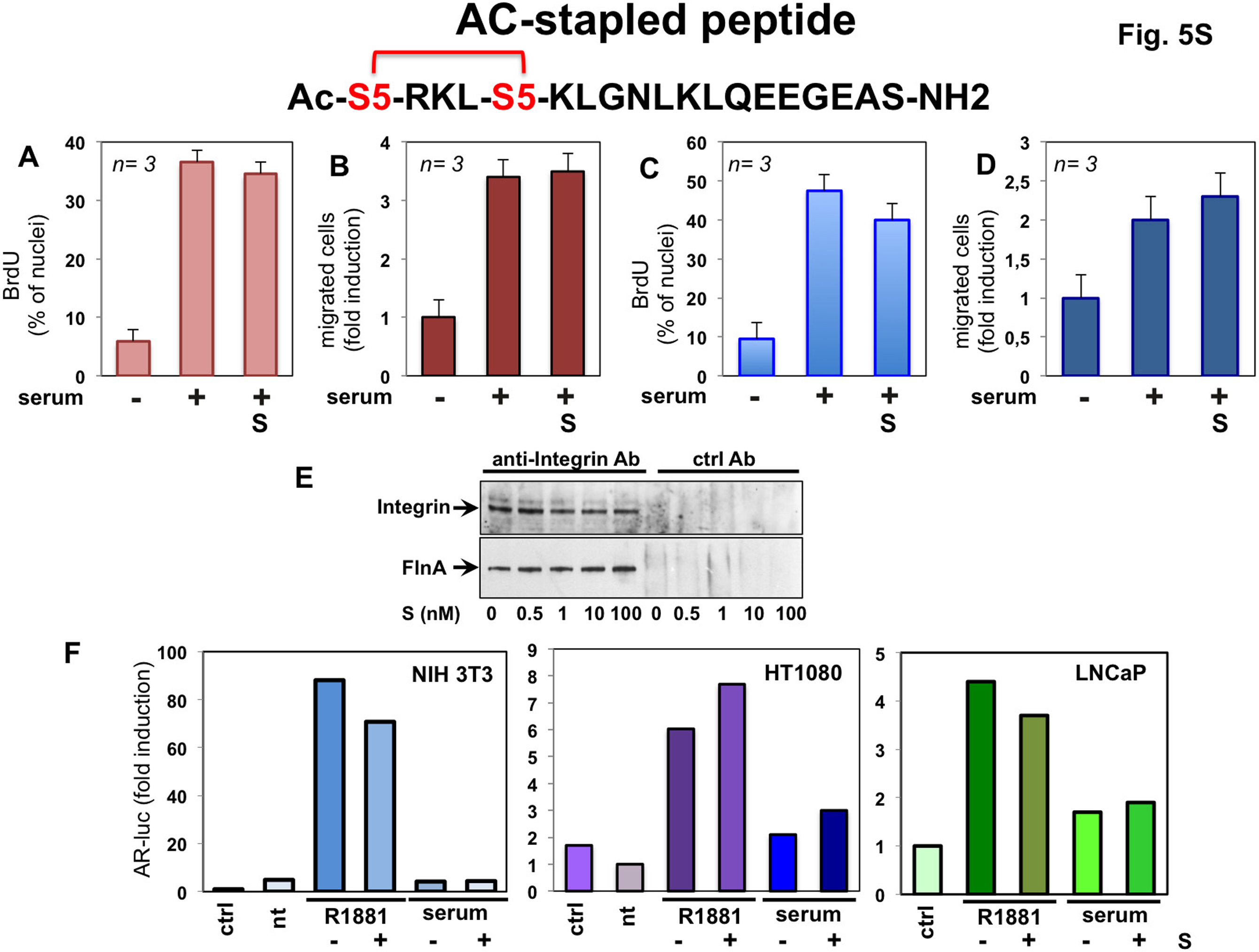

Supplement: Supplementary Figure S5 [file cddis2014497x5.tif]

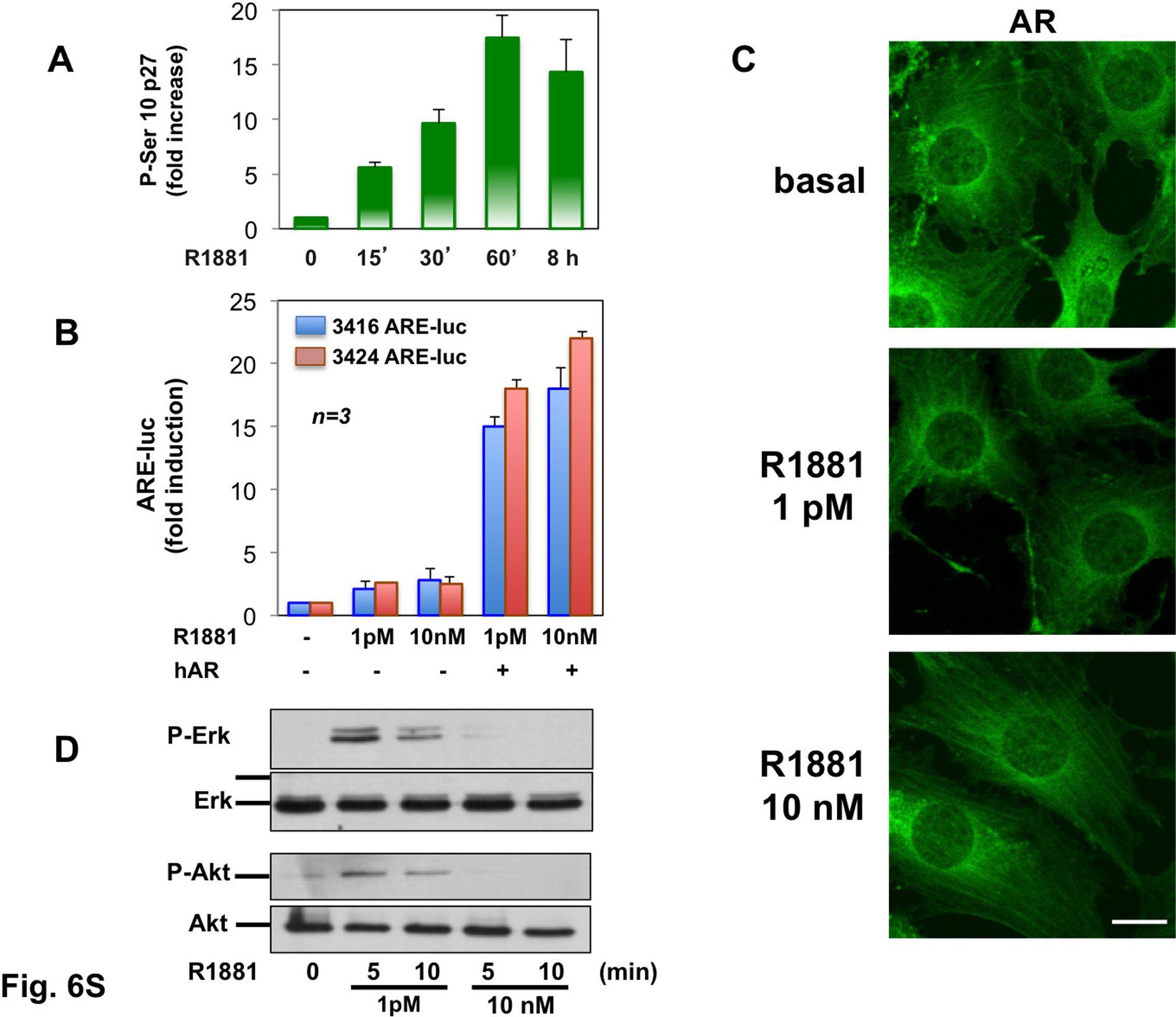

Supplement: Supplementary Figure S6 [file cddis2014497x6.tif]
