# Supplementary material for: Role of non-genomic androgen signalling in suppressing proliferation of fibroblasts and fibrosarcoma cells
Source: Cell Death Dis. 2014 Dec 4;5(12):e1548–. doi: 10.1038/cddis.2014.497 (PMC4649827; doi:10.1038/cddis.2014.497)
Supplement: Supplementary Figure Legends [file cddis2014497x7.doc]

**SUPPLEMENTAL DATA**

**Fig. 1S- NIH3T3 fibroblasts do not express ER or PgR and do not respond to estradiol or progestins.**

Results in **A** show that quiescent NIH3T3 fibroblasts do not migrate in Transwell assay upon stimulation with 10 nM E_2_ or R5020, whereas they migrate upon stimulation with 10 nM R1881 (R) or DHT (D), and bicalutamide (Bic) inhibits this response. The effect of low concentration (1 pM) of R1881 (R) or DHT (D) on cell migration is negligible. NIH3T3 fibroblasts do not express ERalpha or PgR, as assessed by Western blot of lysates using appropriate antibodies in **B**. Western blot analysis of lysates from breast (MCF-7 and T47D) cancer-derived cells is shown for comparison. Panels **C** and **D** show that quiescent NIH3T3 cells do not undergo DNA synthesis and do not proliferate upon stimulation with 10 nM R1881, estradiol or progestin R5020, as assessed by BrdU incorporation (**C**) and MTT assay (**D**). Suboptimal concentration (1 pM) of R1881 increases BrdU incorporation (**C**) and *in vitro* growth (MTT assay in **D**). Means and SEM are shown.

**Fig. 2S- Untransformed and transformed fibroblasts express AR and undergo cell migration upon 10 nM R1881 stimulation.**

NIH3T3 cells, MEFs, MFs, Ras- and Src-transformed NIH3T3 cells as well as HT1080 cells express AR, as verified by Western blot of lysates with the C-19 anti-AR Ab (**A** and **B**). Quiescent MEFs (**C**), MFs (**D**) and HT1080 cells (**E**) migrate in Transwell assay upon stimulation with 10 nM R1881. Bicalutamide inhibits this response. As in NIH3T3 fibroblasts, the effect of low (1 pM) R1881 concentration on cell migration is negligible. Means and SEM are shown.

**Fig. 3S- Regulation of p27 by R1881 in NIH3T3 cells.**

NIH3T3 cells were used. In **A**, cells transfected with the p27-luc promoter were made quiescent, and then left untreated or treated for 10 h with the indicated compounds. Serum was added at 20%. Luciferase activity was assayed, normalized using beta-gal as an internal control, and expressed as fold induction.

In **B**, quiescent cells on coverslips were left untreated or treated with R1881 (1 pM or 10 nM). p27 was stained and visualized by IF. Representative images captured after 12 h hormone stimulation are shown. Bar**,** 10 μM**.** In **C**, quiescent cells were untreated or treated for the indicated times with 10 nM R1881. Lysate proteins were analyzed by Western blot, using antibodies against P-Ser10 p27. The corresponding Western blots were analyzed using the NIH Image J program. Bars in the graph represent the relative increase in Ser10 p27 phosphorylation. Means and SEM are shown.

**Fig. 4S A- FACS analysis of androgen-stimulated NIH3T3 cells.**

Cycling (CC) or quiescent NIH3T3 cells were used. Quiescent cells were left unstimulated or stimulated with 10 nM R1881 for the indicated times. Cells were re-suspended and analyzed by FACS, as described in Methods. The table in **A** shows means and SEM from three different experiments.

**Fig. 4S (B and C)- The Rac inhibitor EHT1864 blocks cell migration induced by 10 nM R1881 in NIH3T3 and HT1080 cells.**

Quiescent NIH3T3 (**B**) or HT1080 (**C**) cells were left unstimulated or stimulated with 10 nM R1881, in the absence or presence of EHT1864 (10 μM), or bicalutamide (Cx, 10 μM). Control cells were treated with EHT1864 alone. Cells were allowed to migrate in Trans-well chambers. Migrated cells were scored by fluorescent microscopy and data expressed as relative increase. Means and SEM are shown.

**Fig. 5S- Properties of the stapled peptide interfering in AR/FlnA complex assembly.**

The sequence of AC-stapled peptide is shown. Quiescent NIH3T3 (**A** and **B**) and AR-negative DU-145 (**C** and **D**) cells were left untreated or treated for 18 h (**A** and **C**) or 6 h (**B** and **D**) with 20% serum, in the absence or presence of S peptide (10 nM). BrdU incorporation (**A** and **C**) and migration (**B** and **D**) assays were performed and represented as described in the main text. In **E**, lysate proteins from NIH3T3 cells were prepared and immunoprecipitated using the anti-beta 1 integrin antibody (anti- integrin Ab). Control lysates were immunoprecipitated with a non-specific IgG (ctrl Ab). Immune complexes were analyzed using the antibodies against the indicated proteins. In **F**, NIH3T3, HT1080 or LNCaP cells were transfected with ARE-luc 3416 reporter gene. Since NIH3T3 and HT1080 cells harbour a transcriptionally incompetent AR, the hAR encoding plasmid was included in transfection. Cells were made quiescent, and then left unstimulated or stimulated with 10 nM R1881 or 20% serum, in the absence or presence of 10 nM S peptide. Luciferase activity was assayed, normalized using beta-gal as an internal control, and expressed as fold induction. The graph shows data from two different experiments. In **A-D**, means and SEM are shown.

**Fig. 6S A- p27 Ser10 phosphorylation by androgens in Ras-transformed fibroblasts.**

Ras-transformed cells were maintained for three days in 0.5% dextran-coated charcoal-stripped serum and then left unstimulated or stimulated for the indicated times with 10 nM R1881. Lysate proteins from three different experiments were analyzed using the anti-P-Ser10 p27. The corresponding Western blots were analyzed using the NIH Image J program. Bars in the graph represent the relative increase in p27 Ser10 phosphorylation. Means and SEM are shown.

**Fig. 6S (B-D)- NIH3T3 cells harbour a transcriptionally incompetent AR, which is localized outside nuclei and activates signalling effectors depending on ligand concentration.**

NIH3T3 cells were used. In **B**, cells were transfected with ARE-luc 3416 or 3424 reporter gene, in the absence or presence of hAR encoding plasmid. Cells were made quiescent and then left unstimulated or stimulated with 10 nM R1881. Luciferase activity was assayed, normalized using beta-gal as an internal control, and expressed as fold induction. In **C**, quiescent cells on coverslips were left unchallenged or challenged for 60 min with the indicated concentrations of R1881. AR was visualized by IF and images were captured by confocal microscope. Bar, 5μM. In **D**, quiescent cells were left unchallenged or challenged for the indicated times with R1881 (1 pM or 10 nM). Lysate proteins were immune-blotted using the antibodies against the indicated proteins.
